# Preclinical In Vitro Assessment of Submicron-Scale Laser Surface Texturing on Ti6Al4V

**DOI:** 10.3390/ma13235342

**Published:** 2020-11-25

**Authors:** Luiz Schweitzer, Alexandre Cunha, Thiago Pereira, Kerstin Mika, Ana Maria Botelho do Rego, Ana Maria Ferraria, Heinz Kieburg, Sven Geissler, Eckart Uhlmann, Janosch Schoon

**Affiliations:** 1 Fraunhofer Institute for Production Systems and Design Technology, Pascalstr. 8-9, 10587 Berlin, Germany; Eckart.Uhlmann@iwf.tu-berlin.de; 2 Department of Orthopedics and Orthopedic Surgery, University Medicine Greifswald, 17475 Greifswald, Germany; Janosch.Schoon@med.uni-greifswald.de; 3 SENAI Innovation Institute in Manufacturing Systems and Laser Processing, Rua Arno Waldemar Döhler 308, Joinville, 89218-153 Santa Catarina, Brazil; alexandre.cunha@sc.senai.br (A.C.); thiago.pereira@sc.senai.br (T.P.); 4 Genetoo Inc., 9841 Washingtonian Blvd, Suite 200, Gaithersburg, MD 20878, USA; 5 Julius Wolff Institute, Charité Universitätsmedizin Berlin, Augustenburger Platz 1, 13353 Berlin, Germany; kerstin.mika@charite.de (K.M.); sven.geissler@charite.de (S.G.); 6 Berlin Institute of Health Center for Regenerative Therapies, Charité Universitätsmedizin Berlin, Augustenburger Platz 1, 13353 Berlin, Germany; 7 BSIRG, Departamento de Engenharia Química, iBB-Institute for Bioengineering and Biosciences, Instituto Superior Técnico, Universidade de Lisboa, 1049-001 Lisboa, Portugal; amrego@tecnico.ulisboa.pt (A.M.B.d.R.); ana.ferraria@tecnico.ulisboa.pt (A.M.F.); 8 Laser-Mikrotechnologie Dr. Kieburg, James-Frank-Str. 15, 12489 Berlin, Germany; dr.kieburg@laser-mikrotech.de; 9 Institute for Machine Tools and Factory Management, Technische Universität Berlin, Pascalstr. 8-9, 10587 Berlin, Germany

**Keywords:** Ti6Al4V, laser surface texturing, LIPSS, biocompatibility, cytotoxicity

## Abstract

Loosening of orthodontic and orthopedic implants is a critical and common clinical problem. To minimize the numbers of revision surgeries due to peri-implant inflammation or insufficient osseointegration, developments of new implant manufacturing strategies are indicated. Ultrafast laser surface texturing is a promising contact-free technology to modify the physicochemical properties of surfaces toward an anti-infectious functionalization. This work aims to texture Ti6Al4V surfaces with ultraviolet (UV) and green (GR) radiation for the manufacturing of laser-induced periodic surface structures (LIPSS). The assessment of these surface modifications addresses key aspects of topography, morphology and chemical composition. Human primary mesenchymal stromal cells (hMSCs) were cultured on laser-textured and polished Ti6Al4V to characterize the surfaces in terms of their in vitro biocompatibility, cytotoxicity, and metal release. The outcomes of the in vitro experiment show the successful culture of hMSCs on textured Ti6Al4V surfaces developed within this work. Cells cultured on LIPSS surfaces were not compromised in terms of their viability if compared to polished surfaces. Yet, the hMSC culture on UV-LIPSS show significantly lower lactate dehydrogenase and titanium release into the supernatant compared to polished. Thus, the presented surface modification can be a promising approach for future applications in orthodontics and orthopedics.

## 1. Introduction

Inflammations of peri-implant tissues are most induced by infection with bacteria. In orthopedic surgery, periprosthetic joint infection (PJI) remains a devastating complication [1]. In dental medicine, peri-implantitis is described as an inflammatory process affecting the tissues surrounding the dental implants, at various degrees of severity [2]. It is expected that the increasing use of implants in medicine will lead to a natural increase in the number of associated infections. Furthermore, peri-implant infections can manifest in severe pain symptomatic and are associated with a significantly increased risk for bone loss and severe concomitant diseases including cardiovascular events and the acute onset of systemic infection [3,4]. In a previous study our research group investigated the effects of laser surface nanotexturing for the reduction of peri-implantitis on Ti-6Al-4V dental abutments [5]. The outcomes of the study show a significant reduction of biofilm formation from the early colonizer *S.salivarius* on the surface. However, new developments in implantology must be assessed from all angles. Beside the focus on the inhibition of post-operative biofilm formation, an unhindered foreign body reaction and rapid regeneration of the surrounding tissues should be additionally addressed in early pre-clinical investigations [6,7,8].

In orthopedic, maxillofacial, and dental surgery the application of metals, ceramics, or polymers is widely established [9]. Commonly used metals include titanium and its alloys, cobalt-chromium-molybdenum, and tantalum [10]. Various types of titanium alloys were successfully developed and produced for various medical applications [11]. Titanium-aluminum-vanadium alloy (Ti6Al4V) is frequently used for weight bearing but non-articulating implant components and became well-known in the 1950s because of beneficial physical and chemical properties [12].

The implant’s topography influences the cellular response and thus the performance of the implant [13]. Consequently, several techniques on surface modification of titanium were introduced. Conventional techniques such as sandblasting [14,15,16,17,18], chemical etching [16,17,18], and coatings [17] were applied. In addition, laser treatment is a more recent technology with several advantages including a reduced risk for surface contamination [19] and the possibility of producing stochastic or precise deterministic structures in micro and nanometer ranges [20]. Several studies have shown the potential of ultrashort-pulsed lasers on the structuring of titanium for biomedical implants [5,21,22,23]. This technology is superior to short-pulsed laser sources because of the enhanced flexibility for surface patterning [24] as well as less minimal thermal damage within the pulse duration of 10^−15^ s ≤ t_L_ ≤ 10^−12^ s [25].

Laser-induced periodic surface structures (LIPSS) are self-organized formations observed on the surfaces treated with polarized laser radiation. There are two types of LIPSS, which can be differentiated according to the relation between the spatial periodicity Λ and the radiation wavelength λ. High spatial frequency LIPSS (HSFLs) have a significantly smaller spatial periodicity Λ than the radiation wavelength λ, which ranges only from a quarter to a tenth of the wavelength λ size. The mechanisms for its formation remain controversial, typical examples are the self-organization [26], second-harmonic generation [27], and Mie scattering [28]. Low spatial frequency LIPSS (LSFLs) have spatial periodicity Λ that is only slightly smaller to the wavelength λ. The first theory for its formation considered the interference between the incident laser beam and scattered electromagnetic waves from the surface [29], supported by the theorems proposed by Sipe et al. [30]. Recent studies follow the hypothesis that the laser irradiation interacts with electromagnetic waves associated to the material surface, called surface plasmons polaritons (SPPs). In this model, the interference between the incident laser radiation and the SPPs causes a periodic spatial modulation of the deposited laser energy E, which forms the periodic structures [31].

Independent from anti-infectious properties of an implant material, surface modifications can lead to altered biocompatibility, cytotoxicity, and metal release in vivo. Preclinical testing of implant materials aims to apply in vitro models emulating important cell-mediated consequences in vivo. Human mesenchymal stromal cells (hMSCs), as the precursors of matrix forming soft tissue cells, are not only an important cell source for tissue engineering in regenerative medicine but also valuable for preclinical in vitro biocompatibility testing of implant materials [32,33]. Biocompatibility can be affected by changing the chemical speciation of the surfaces which consequently leads to e.g., altered ingrowth and metal release capacities. Thus, the hypothesis of this work is that the laser surface texturing with ultrafast laser pulses can provide optimized biocompatibility of the implant material. The aim is to manufacture sub-micron scale surface textures on Ti6Al4V to optimize the growth of human cells without the addition of any material to the titanium alloy. To this end, the textured surfaces were characterized and in vitro culture of hMSCs on the surface textures were applied. This approach allows for addressing the impact of morphological and chemical changes on the materials’ biocompatibility. This in vitro assessment of implant modifications is of upmost importance in the process of preclinical testing to ensure maximum patient safety during future implantations.

## 2. Materials and Methods

### 2.1. Material

Ti6Al4V ELI raw material from High Tech Alloys Sonderwerkstoffe GmbH, Wuppertal, was applied for the experiments. It attends the norm ISO 5832-3 [34] concerning the characteristics of the titanium alloy as implant material with common Ti6Al4V chemical composition (Table 1). The samples were manufactured as disks for fitting them into well plates for the in vitro experiments. This sample geometry corresponds to the sample diameter d_s_ = 10.0 mm, sample height h_s_ = 2.0 mm, and sample phase angle δ_s_ = 0.25 × 45.0. The implant manufacturer A.K.TEK Medizintechnik GmbH, Hagen, Germany, produced the samples with the lathe model Deco 13, Tornos S.A., Moutier, Switzerland. As a standard procedure applied by the company, all abutments were electropolished as finishing process to remove debris and provide lower surface roughness. The titanium alloy dental implants-specific electrolyte ElpoLux TI-Med, ElpoChem AG, Volketswil, was applied. Samples were separately treated according to the electrolyte manufacturer instructions and conditions at room temperature ϑ_R_ = 20 °C for the polishing time to = 5.0 min. The polished surface (PO) was applied as reference within the in vitro experiments.

### 2.2. Laser Processing

Two laser machine tools were applied to carry out the laser processing. Both are equipped with the ultrashort pulsed laser source Talisker-Three, Coherent, Santa Clara, USA. It operates within three wavelengths λ_IR_ = 1064 nm, λ_GR_ = 532 nm, or λ_UV_ = 355 nm, pulse duration tL = 10 ps and the maximum pulse frequency f = 500 kHz. The description of the laser machine tools is given as follows: Laser machine tool 1 (LMT1): The model LMBS 3W-015-xy300z200-IA, applied for the UV laser texturing, from Lasermikrotechnologie Dr. Kieburg GmbH, Berlin, Germany. The machine has an average beam power PL = 3.0 W and a laser beam diameter of du = 12.0 μm at the focusing position. Therefore, the third harmonic λ_UV_ = 355 nm was applied for the experiments. Laser machine tool (LMT2): The MJ-Series model from Oxford Lasers Ltd., Didcot-Oxford, UK. The machine has an average beam power PL = 8.0 W and laser beam diameter du = 16.0 μm at the focusing position. The second harmonic λ_GR_ = 532 nm was applied for the experiments.

**Figure 1:**
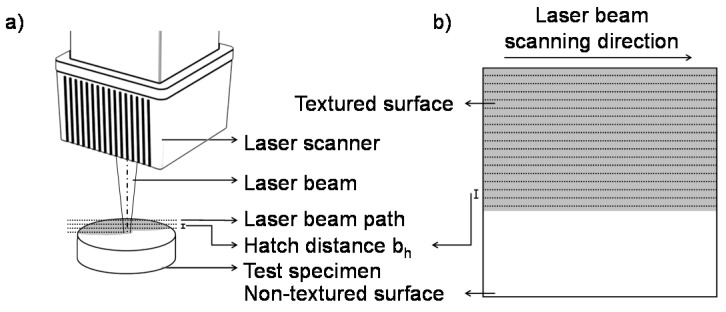
Schematic illustration of the experiments; (**a**) laser set-up to produce textured surfaces; (**b**) surface texturing according to the hatch distance b_h._

The laser surface texturing procedures were performed according to the laser processing methods described by Oliveira [35,36]. The processing technique allows the manufacturing of a specific type of nanotexture, often referred to as LIPSS. The applied laser average fluence Fav was estimated by applying the D-squared method [37]. Displacement of the laser beam was carried out by galvo heads with a scanning area As = 50.0 × 50.0 mm^2^. The laser beam was focused and linearly scanned perpendicularly to the surface. To achieve complete surface coverage the laser beam was laterally displaced by a hatch distance b_h_ to partially overlap the individual laser tracks as presented by Figure 1. The lateral overlap rate α is calculated according to Equation (1).

(1) $$\alpha =\left(1-\frac{b_{h}}{d_{u}}\right)\cdot 100$$

The laser material processing was performed in ambient atmosphere. Table 2 summarizes the laser processing parameters applied to manufacture the LIPSS for each laser system used.

### 2.3. Surface Characterization

The surface morphology was qualitatively assessed by scanning electron microscopy (SEM), with the microscope LEO 1455VP, Zeiss GmbH, Oberkochen, Germany. The SEM micrographs were acquired at the magnification M = 15,000×, under an accelerating voltage V = 15.0 keV and chamber pressure p = 40.0 Pa. The surface topography was quantitatively assessed by atomic force microscopy (AFM), applying the microscope N8 Neos, Bruker CORP, MA, USA. Images with a scan area AA = 10.0 × 10.0 μm^2^ and a resolution R = 1024 × 1024 pixels were acquired using standard silicon (Si) tips. At least five different images were acquired at five different locations of the surfaces for three (n = 3) specimens per condition. The open source software Gwyddion^®^ V2.56 was applied to evaluate the surface roughness and profile.

X-ray photoelectron spectroscopy (XPS) was applied for the chemical characterization of the near-surface regions from the sample. The chemical analysis was performed with the spectrometer XSAM800, Kratos Analytical LTD., Manchester, UK. It was operated with the ALKα anode with X-ray energy hν = 1486.6 eV within ultra-high vacuum pressure p = 10−13 bar at ϑ_R_ = 20 °C. Each spectrum was acquired with a step of ΔBE = 0.1 eV and measuring time tm = 60 s by sweep, applying the software Vision 2 for Windows, Version 2.2.9 from Kratos Analytical LTD., Manchester, UK. The binding energy (BE) was corrected considering the charge shift observed for the sp3 C 1s peak from C–C and C–H that should be centered at hν = 285 eV. The peak fitting was performed with the freeware XPSPeak 4.1. The assignment of the peak components was mainly based on the X-ray photoelectron spectroscopy database of the National Institute of Standards and Technology (NIST), Gaithersburg [38]. For quantitative purposes, the following sensitivity factors were used: Ti 2p: 2.001, Al 2p: 0.193, and V 2p: 1.444.

The assessment of the surface wettability was realized by the sessile drop method with distilled-deionized water as target liquid. The liquid droplets, with a volume V_w_ = 100.0 μL, were deposited on the specimen’s surface by using a micrometric syringe. The analyses were performed at room temperature ϑR = 20 °C. Several images of the droplets were acquired in the timeframe td ≤ 1 s with an integrated camera-system perpendicularly oriented to the laser beam scanning direction. The contact angle θ was determined by the axisymmetric drop shape analysis profile (ADSA-P) method, using a Drop Shape Analyzer 100 (DSA), Krüss GmbH, Hamburg, Germany. A regression analysis of the measured contact angle *θ* raises information about the drop-spreading kinetics and therefore the fluid film-spreading capacity. The fitting follows Equation (2), in which *k* and the spreading coefficient *η* are constants prevenient from experimental data [39].

*θ* = *k* · *t^η^* (2)

### 2.4. Cell Culture

Human MSCs were received from the Core-Facility “Cell Harvesting” of the BIH Center for Regenerative Therapies (BCRT). Cells were isolated from the bone marrow biopsy of a female 64-years-old patient undergoing hip replacement at Charité University Hospital, as previously described by Rakow et al. [40]. Written informed consent was given, and ethics approval was obtained from the Institutional Review Board (IRB) of the Charité-Universitätsmedizin Berlin (IRB approval EA1/194/13). The cells were cultured at the incubation temperature ϑ_i_ = 37 °C in Dulbecco’s modified Eagle’s medium with low glucose, Sigma Aldrich Inc., St.Louis, MO, USA and supplemented with fetal calf serum (FSC) φ_F_ = 10%, Biochrom GmbH, Berlin, Germany, GlutaMAXTM φ_G_ = 1%, Thermo Fischer Scientific Inc., Waltham, MA, USA, and penicillin/streptomycin φ_P_ = 1%, Carl Roth Gmbh&Co. Kg, Karlsruhe, Germany, for all in vitro experiments. The cells were sub-cultured in cell passages from 3 ≤ C_s_ ≤ 4. Only the osteogenic differentiation was cultured with cell confluency C_c_ = 80%, the other in vitro experiments were conducted with C_c_ = 30%. Cell metabolic activity, cell proliferation, lactate dehydrogenase (LDH) release, metal release, and cell differentiation were performed in individual experiments. Cell culture on laser-textured surfaces was realized in 48-well non-tissue culture treated plates, Sigma-Aldrich Inc., St.Louis, MO, USA. The titanium samples were press fitted in the wells, parallel to the surface to provide an equal distribution of supernatant over the entire surface.

### 2.5. Cell Metabolic Activity

The resazurin-based calorimetric agent PrestoBlueTM, Thermo Fischer Scientific Inc., Waltham, MA, USA, indicates cell viability by quantifying the cell metabolic activity C_m_. Metabolic activity was measured at day three, five, seven, and nine after cell seeding. A volume V_x_ = 200 µL was applied for each well containing a single sample at the incubation temperature ϑ_i_ = 37 °C for the incubation time t_i_ = 1 h. Fluorescence intensity I_f_ was measured at the excitation wavelength λ_ex_ = 560 nm and emission wavelength of λ_em_ = 590 nm in the multimode microplate reader Infinite M200 Pro, Tecan Group Ltd., Männerdorf, Germany.

### 2.6. Cell Proliferation

The cell proliferation was determined applying CyQUANT, Thermo Fischer Scientific Inc., Waltham, UK, according to the manufacturer’s protocol. The cell proliferation was performed after one, four, and eight days after MSC seeding at cell density C_c_ = 30%. A longer culture period is not indicated because of the cell–cell contact-induced inhibition of proliferation after the cell layer reaches full confluency. The wells were washed with phosphate buffered saline (PBS), Biochrom GmbH, Berlin, Germany and frozen at temperature ϑ_f_ = −80 °C for the incubation time t_i_ = 12 h. The assay was realized by incubation of the dye volume V_y_ = 150 µL at room temperature ϧ_R_ = 20 °C for the dye incubation time t_i_ = 5 min. The fluorescence intensity I_f_ was measured at the excitation wavelength λ_ex_ = 485 nm and emission wavelength λ_em_ = 530 nm.

### 2.7. LDH Release

The LDH release CL quantification was realized by application of the cytotoxicity detection KitPLUS (LDH), Sigma-Aldrich Inc., St.Louis, MO, USA according to the manufacturer’s instructions with minor modifications. LDH quantification was conducted at t_c_ = 3 days, t_c_ = 8 days, and t_c_ = 15 days after seeding. In brief, the cell supernatant volume V_u_ = 25 µL was mixed with reagent volume V_x_ = 25 µL at room temperature ϧ_R_ = 20 °C for the reaction time t_x_ = 15 min. After this, a stop solution V_x_ = 12.5 µL was added into the solution for halting the reaction. The absorption was measured at the measuring wavelength λ_m_ = 492 nm and reference wavelength λ_re_ = 690 nm.

### 2.8. Metal Release

Quantification of the multiple metals was realized by inductively coupled plasma mass spectrometry (ICP-MS). To this end, the supernatant volume V_u_ = 150 µL was collected from each media change and cumulatively stored to analyze the total metal release during the whole exposure period. Cells were cultured for eight days. The media was collected at day two (54 h after seeding), five, seven, and eight. To assess the release of titanium (Ti), and vanadium (V), the supernatant was chemically digested by V_s_ = 3 mL nitric acid (HNO_3_). For the analysis of aluminum (Al) and iron (Fe), the supernatant was chemically digested by V_s_ = 3 mL hydrochloric acid (HCl). The quantification was realized with ICapQ, Thermo Fischer Scientific Inc., Waltham, MA, USA in collision reaction mode.

### 2.9. Osteogenic Differentiation

The cells’ capacity for osteogenic differentiation was assayed by alkaline phosphatase (ALP) activity C_A_ and mineral matrix content C_M_. The ALP activity C_A_ was quantified as previously shown [41] by incubation with the substrate 4-nitrophenylphosphate (pNPP) and subsequent measurement of absorption six days after seeding (five days after osteogenic stimulus).

The assessment of the cell mineral matrix content C_M_ was realized by calcium staining with Alizarin RedS, Sigma-Aldrich Inc., St.Louis, MO, USA, and subsequent extraction with cetylpyridiniumchlorid solution and measurement of the absorption 17 days after seeding (t_c_ = 16 days after osteogenic stimulus) as previously described [42].

### 2.10. Statistical Analysis

The results presented correspond to an average value and its standard deviation. The statistical analysis applied for this work was the one-way analysis of variance (ANOVA) and Tukey’s test for multiple comparisons. Differences were considered statistically significant if the p-value was p < 0.05 (*) or p < 0.01 (**), which correspond to a confidence of 95% or 99% respectively.

## 3. Results and Discussion

### 3.1. Laser Surface Texturing

The surface structures manufactured in the present work are low-spatial frequency LIPSS (LSFLs) produced by radiation wavelength λ = 355 nm (UV-LIPSS) and λ = 532 nm (GR-LIPSS). The ripples from LIPSS structures are perpendicular to the polarization direction of the linearly polarized laser beam.

#### 3.1.1. Surface Morphology and Topography

**Figure 2:**
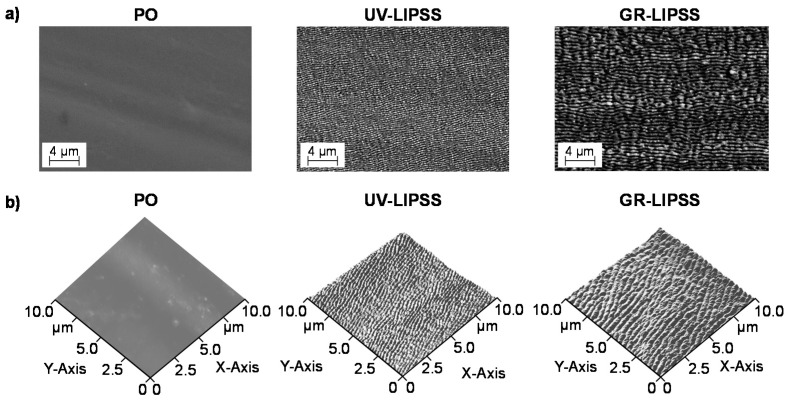
Surface characteristics of polished, UV-LIPSS, and GR-LIPSS. (**a**) SEM micrographs of Ti6Al4V surfaces. (**b**) AFM topography of Ti6Al4V surfaces.

SEM micrographs indicate the periodic texturing of the surfaces (Figure 2a). The AFM measurement enables for depicting a 3D-view of the surface topography of PO, UV-LIPSS, and GR-LIPSS (Figure 2b). Table 3 summarizes the surface characteristics by means of arithmetical mean height Sa, maximum height Sz, skewness Ssk, kurtosis Sku, and profile height h_P_. For both Sa and Sz, the laser processing leads to significantly higher outcomes than the polished surface. Although the manufacturing of surface textures increases the Sa and Sz roughness, the processing within ultraviolet radiation leads to lower outcomes than green radiation. This may be justified by the average fluence Φ_a_, which corresponded to 40% of that applied for the GR-LIPSS. Furthermore, the ultraviolet radiation also comprises a smaller beam waist radius ω_0_ that reduces the surface roughness. Taken together, the surface texturing resulted in low spatial frequency LIPSS (LSFLs), which present spatial periodicity Λ slightly inferior to the radiation wavelength λ. The processing with ultraviolet manufactured UV-LIPSS with spatial periodicity of 210 nm ≤ λ ≤ 274 nm while the green radiation resulted in 404 nm ≤ λ ≤ 492 nm (Figure 2b).

The skewness Ssk corresponds to the symmetry of the height distribution and characterizes the morphology of the surface structures. Values close to zero indicate a symmetric profile, which in the case of LIPSS corresponds to the continuity of ripples on the entire surface [43]. The skewness Ssk results show significant differences between PO and the textured surfaces. However, both UV-LIPSS and GR-LIPSS are symmetric with profiles resulting in 0.03 ≤ Ssk ≤ 0.17 and 0.07 ≤ Ssk ≤ 0.29 respectively. The mean value close to zero and low variance resemble to the Gaussian profile with Ssk = 0. The kurtosis Sku provides information on the sharpness of the height distribution. Surfaces with Sku > 3 consist of sharp peaks, whereas Sku < 3 reveals rounded peaks. For all the samples treated with ultraviolet and green radiation, the kurtosis Sku results were lower than 3 indicating the presence of rounded peaks. A Gaussian surface presents skewness Ssk = 0 and kurtosis Sku = 3 [43]. 

#### 3.1.2. Chemical Characterization

**Figure 3:**
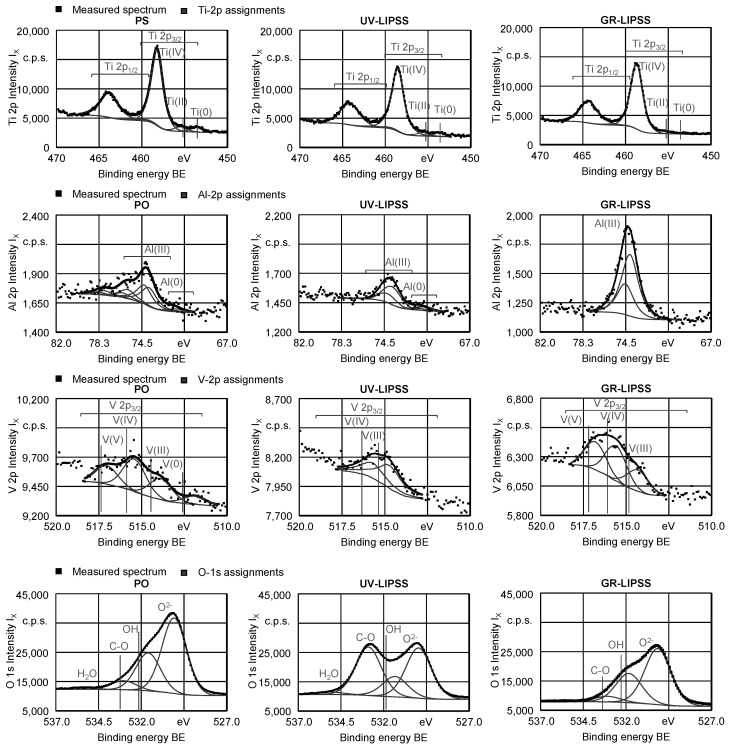
X-Ray photoelectron spectra for regions Ti 2p, Al 2p, V 2p_3/2_, and O 1s of PO, UV-LIPSS and GR-LIPSS.

The surface characteristics in terms of chemical speciation was analyzed by XPS (Figure 3) for Ti, Al, V, and O. In Ti 2p three doublets were fitted with a spin-orbit separation around BE = 5.7 eV; the main components of each doublet, Ti 2p_3/2_, are positioned at 458.3 eV ≤ BE ≤ 458.7 eV, assigned to Ti (IV) in TiO_2_ at 455.4 eV ≤ BE ≤ 455.6 eV, assigned to Ti (II) in TiO, and at 453.5 eV≤ BE ≤ 453.7 eV, assigned to Ti (0), corresponding to metallic titanium. In the case of aluminum, several doublets were fitted and the most intense with the main component, Al 2p_3/2_, centered at 73.7 eV ≤ BE ≤ 74.1 eV, is assigned to Al (III) in Al_2_O_3_. The V 2p region is fitted with several doublets the most intense being the one assigned to V (IV) with the main component centered at 515.4 eV ≤ BE ≤ 515.6 eV. The O 1s region for the Ti6Al4V samples was fitted with four components except for the GR sample where just three were enough. The 529.9 eV < BE < 530.1 eV is assigned to oxide (O^−2^); the 531.3 eV < BE < 531.8 eV component to a mixture of hydroxyl (OH) and carbonyl groups (C=O); the 532.9 eV < BE < 533.1 eV component to oxygen singly bound to carbon (C-O); and finally, at 535 eV < BE < 535.4 eV a component assigned to H_2_O occluded in the samples where it appears.

The XPS intensity I_X_ shown in the spectra of Ti 2p, Al 2p, V 2p, and O 1s, follow a trend.

The quantitative analysis for the metallic elements (Table 4) shows that no metallic forms are detected in GR-LIPSS sample showing that the oxidized layer thickness is b_o_ ≥ 10 nm. The average photoelectron escape depth for the analyzed peaks around b = 2 nm and, therefore, more than 99 % of the signal comes from a depth five times larger. Beyond that depth no measurable signal is obtained. The oxide layer of GR-LIPSS is thicker than the other ones and the first O 1s peak at BE = 533 eV shows formation of C-O groups on the UV-LIPSS surface. Furthermore, the stoichiometry of the surface, in terms of the metallic species is different to the bulk material. Textured surfaces are richer in titanium species than in aluminum or vanadium, in which the expected ratio is of 1.5 and 6.0 respectively. The following rough stoichiometries can be extracted: Ti_26_Al_6.8_V (PO), Ti_32.2_Al_5.8_V (UV-LIPSS), and Ti_20_Al_11_V (GR-LIPSS).

Titanium (Ti) is a highly reactive material, which reacts with oxygen (O) by simple exposure to air the laser radiation changing its extent [44]. Therefore, even the polished surfaces presented an oxide layer on the surface. The surface treatment induces rearranging and modification of the existent oxide layer, which is related to the high temperatures ϑ reached during the laser processing [45]. The presence of a TiO_2_ layer suggests higher biocompatibility and corrosion resistance at physiological pH values because of the low electronic conductivity and thermodynamically stable nature [46]. Furthermore, it supports the incorporation of mineral ions such as calcium phosphates and water, promoting the biological environment to foster the mineralization [47].

#### 3.1.3. Wettability

The sessile drop method defines wetting characteristics by measuring the contact angle θ resulting from the contact between the water droplet and the surface. The surface wettability of PO, UV-LIPSS, and GR-LIPSS show a hydrophilic behavior (Table 5). It shows a reduction of the contact angle θ during the measuring time t_m_, which stabilized for t_m_ ≤ 150 s. The hydrophilicity increases by the measurement perpendicular to the ripples direction, resulting in a difference of 7% and 10% for UV-LIPSS and GR-LIPSS, respectively. The spreading coefficient η of UV-LIPSS is higher than PO and GR-LIPSS. The water droplet resulted in faster spreading corresponding to |η| = 0.22 for UV-LIPSS and |η| = 0.18 for GR-LIPSS. Studies show that the spreading coefficient generally comprises the range of 0.04 ≤ |η| ≤ 0.20 [39,47,48], which approximates to the values obtained for Ti6Al4V within this work. The LIPSS texturing is anisotropic presenting a faster wetting of the surface following the ripples direction. Chung [49] and Zhao [50] reported the same effect on textured surfaces. The adsorption of biological fluids such as water, blood, saline, and protein solutions on the implant surface is a key factor in cell adhesion and differentiation in the early phase of osteoblasts formation [51].

### 3.2. Asessment of In Vitro Biocompatibility

The in vitro experiments involve the evaluation and assessment of cellular responses to the surface modifications produced by the laser texturing technique established through ultraviolet and green radiation. Along the textured and polished Ti6Al4V samples, tissue culture plastic (TCP) is exposed to the same in vitro conditions. The culture on TCP serves as a reference to assess whether the human cells show the expected behavior under the in vitro cell culture and thus validate the experimental results. Therefore, primary human cells were cultivated in direct contact with the surfaces TCP, PO, UV-LIPSS, and GR-LIPSS. The evaluation of the in vitro experiments considers the analyses of cell viability, cell proliferation, lactate dehydrogenase (LDH) released from damaged cells and the release of metallic degradation products into the culture supernatant. At last, the osteogenic differentiation of TCP, PO, and GR-LIPSS is investigated.

#### 3.2.1. Cell Viability

**Figure 4:**
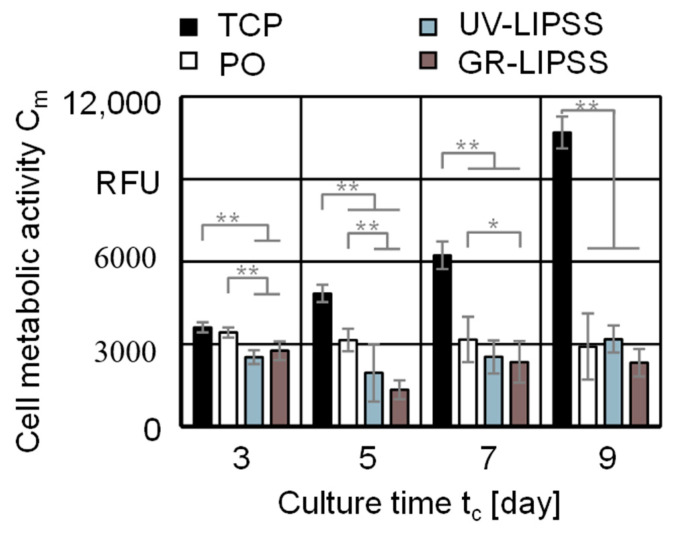
Cell metabolic activity C_m_ on tissue culture plastic (TCP), PO, UV-LIPSS, and GR-LIPSS. Statistical analysis by ANOVA and Tukey’s test for determination of statistically significant differences (n = 5 each group, * for p < 0.05, ** for p < 0.01).

The cell viability assesses the cell metabolic activity C_m_ of hMSCs cultured on TCP, PO, UV-LIPSS, and GR-LIPSS for a culture time t_c_ = 9 days (Figure 4). The metabolic activity of the cells cultured on TCP is significantly higher than the Ti6Al4V surfaces, despite at t_c_ = 3 days compared to PO. The TCP outcomes are significantly higher than the metal surfaces at t_c_ = 7 days and t_c_ = 9 days. The cell culture on PO shows higher C_m_ than the textured surfaces at t_c_ = 3 days. At t_c_ = 5 days it is only higher than GR-LIPSS. By the end of the cell culture, all three metal surfaces present no difference regarding the cell metabolic activity C_m_.

**Figure 5:**
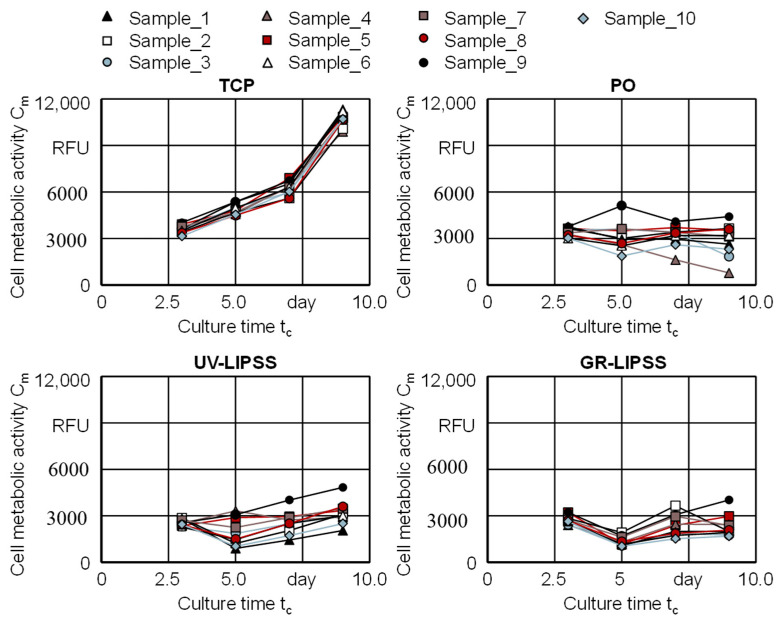
Cell metabolic activity C_m_ for TCP, PO, UV-LIPSS, and GR-LIPSS on each sample individually.

The same experiment is displayed according to the response of each individual sample during the culture time t_c_ = 9 days (Figure 5). The culture on TCP shows both the higher metabolic activity and the lower standard deviations σ if compared to the metal surfaces. There is a constant increase between t_c_ = 3 days and t_c_ = 7 days, presenting a considerable rise at t_c_ = 9 days. The cells on PO have a constant metabolic activity along the culture period. Over UV-LIPSS, the metabolic activity decreases between 3 days ≤ t_c_ ≤ 5 days and rises for the culture time t_c_ ≥ 5 days. The cell culture on GR-LIPSS follows the same pattern, although presenting lower cell metabolic activity C_m_ than UV-LIPSS.

The outcomes show that the cell viability is similar for the metal surfaces, despite of the surface finishing. The experiment revealed that there is a great difference between TCP and Ti6Al4V for the hMSCs. Although the cell viability is not significantly increased with the laser treatment after t_c_ = 9 days, it shows a linear ramp-up for all UV-LIPSS samples and also a slight increase for GR-LIPSS. The same does not occurs with PO, on which the cells have the viability stagnated or reducing over the culture time t_c_.

Gnilitskyi et al. [21] studied the impact of femtosecond laser surface texturing on Ti6Al4V and zirconium (Zr) implants. The study investigated the cell response to LIPSS surface texturing produced by near-infrared laser radiation (NIR-LIPSS) on Human Dermal Fibroblasts-Adult cell lines. The cell viability on both materials was significantly higher with NIR-LIPSS compared to polished surfaces. In this work, the metabolic activity C_m_ for polished and textured surfaces are not significantly different. A substantial difference between the results obtained by Gnilitskyi et al. [21] and this work is the LIPSS spatial periodicity Λ. The NIR-LIPSS yielded a spatial periodicity of 758 nm ≤ Λ ≤ 842 nm, corresponding to 179% of the GR-LIPSS spatial periodicity Λ and 331% of the UV-LIPSS. However, it is mostly unlikely that only the spatial periodicity Λ caused the increase in cellular metabolism because there are no significant differences between UV-LIPSS and GR-LIPSS. Differences in chemical composition of the material surface and different type of cells studied can play an important role in this aspect.

#### 3.2.2. Cell Proliferation

**Figure 6:**
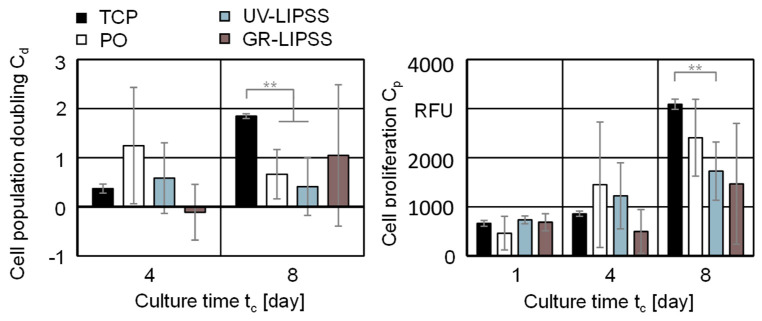
Cell population doubling C_d_ and cell proliferation C_p_ on TCP, PO, UV-LIPSS, and GR-LIPSS. Statistical analysis by ANOVA and Tukey’s test for determination of statistically significant differences (n = 5 each group, ** for p < 0.01).

The cell proliferation C_p_ and cell population doubling C_d_ assess the hMSCs ability to reproduce on TCP, PO, UV-LIPSS, and GR-LIPSS (Figure 6). The cell population doubling C_d_ on TCP is significantly higher than PO and UV-LIPSS at t_c_ = 8 days. The culture on GR-LIPSS show samples with cell population doubling C_d_ similar to TCP, but the high standard deviations σ indicate a reduction of cell population on some samples. Cells on PO and UV-LIPSS present mostly positive values at t_c_ = 8 days, which indicates an increase of the number of cells N_c_ in comparison with the culture time t_c_ = 4 days. The positive outcomes of cell population doubling C_d_ after the culture time t_c_ = 8 days shows that the cells are capable to replicate on all investigated surfaces. The results of cell proliferation C_p_ are not significantly different for t_c_ = 1 day and t_c_ = 4 days. At t_c_ = 8 days, the proliferation on TCP is significantly higher than on UV-LIPSS.

The cell proliferation C_p_ and cell population doubling C_d_ outcomes show low standard deviation σ for the culture on TCP and are comparability high for the metal surfaces. In particular, PO and GR-LIPSS show a higher dispersion of samples than UV-LIPSS. However, there is no significant difference between the metal surfaces investigated. Cunha [47] investigated the laser surface texturing with near-infrared radiation (IR) wavelength λ = 1030 nm and t_L_ = 500 fs on Ti6Al4V. In this study, the NIR-LIPSS resulted in spatial periodicity of 649 nm ≤ Λ ≤ 767 nm and increased the proliferation of hMSCs in comparison with polished surfaces after t_c_ = 14 days.

#### 3.2.3. LDH Release

**Figure 7:**
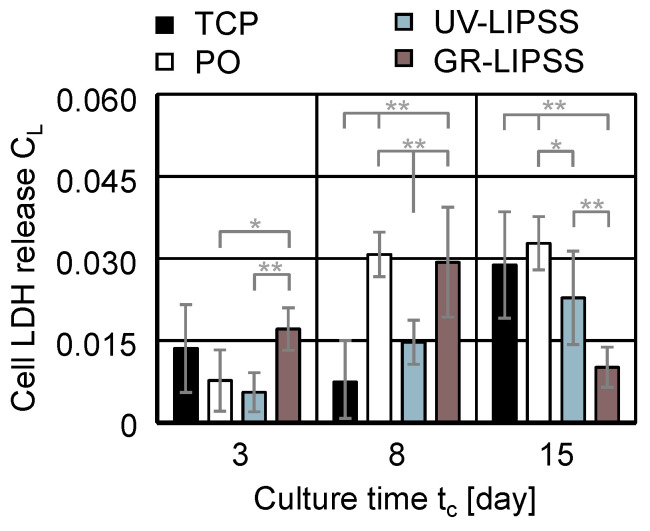
Cell lactate dehydrogenase (LDH) release C_L_ on TCP, PO, UV-LIPSS, and GR-LIPSS. Statistical analysis by ANOVA and Tukey’s test for determination of statistically significant differences (n = 10 each group, * for p < 0.05, ** for p < 0.01).

The lactate dehydrogenase (LDH) corresponds to the cytoplasmic enzyme present inside the cell wall. After the cell death, the cell membrane collapses and the LDH is released into the supernatant. Therefore, the measurement of cell LDH release C_L_ indicates the death of cells within the culture. The assessment included the culture on TCP, PO, UV-LIPSS, and GR-LIPSS during the culture time t_c_ = 15 days (Figure 7). The outcomes for the culture on UV-LIPSS and PO are significantly lower than on GR-LIPSS by t_c_ = 3 days. At t_c_ = 8 days, the cells on TCP and UV-LIPSS show significantly lower cell LDH release C_L_ than PO and GR-LIPSS. At the culture time t_c_ = 15 days, the outcomes on UV-LIPSS and GR-LIPSS are significantly lower than PO. Furthermore, the LDH release of cells cultured on GR-LIPSS is significantly lower than UV-LIPSS and TCP. The results indicate reduced cellular death in UV-LIPSS, presenting outputs comparable to TCP. Vaithilingam et al. [52] investigated the effects of Ti6Al4V surface topography on LDH release C_L_. The study aimed at comparing samples produced by selective laser melding (SLM) process and polished surfaces. The in vitro tests accounted for the exposure of immortalized cells from NIH 3T3 embryonic mouse fibroblasts to the samples. The release of LDH after t_c_ = 1 day and t_c_ = 3 days showed no significant difference. This suggests that although the SLM technology is presented as a viable method for manufacturing biomedical implants, it presents disadvantages in comparison with the laser surface texturing proposed in this work.

#### 3.2.4. Metal Release

**Figure 8:**
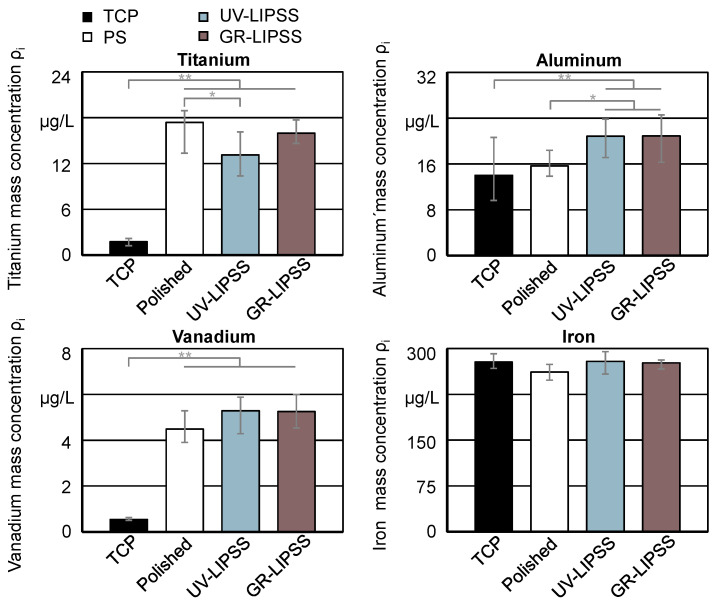
Metal release to supernatant on TCP, PO, UV-LIPSS, and GR-LIPSS after culture time t_c_ = 8 days. Statistical analysis by ANOVA and Tukey’s test for determination of statistically significant differences (n = 5 each group, * for p < 0.05, ** for p < 0.01).

Quantifying the metal released into the supernatant is a major aspect of possible cytotoxic effects due to surface texturing. Results show the metal mass concentration ρi of titanium (Ti), aluminum (Al), vanadium (V), and iron (Fe) in the cumulative culture media after a culture time t_c_ = 9 days (Figure 8). The quantified metal levels in the supernatants of TCP depict the background metal levels of the culture media. Therefore, the TCP results serve as a reference for the assessment of the polished and textured surfaces. The analysis comprises the three main constituents of the Ti6Al4V samples and Fe, which may also be present in the titanium alloy. The results show significantly lower Ti mass concentration ρ_i_ on UV-LIPPS, compared to PO. In the case of Al, the culture on textured surfaces present higher mass concentration ρ_i_ than on PO and TCP. The culture on polished and textured Ti6Al4V show similar results for V. The outcomes for Fe are similar within all four conditions, which indicate no significant difference between the surfaces. Although the technology corresponds to a contact-free manufacturing and does not have the addition of any material, it influences the reactivity of the surface. In the present study, it significantly reduces the release of Ti and increases the release of Al.

The significantly higher mass concentration ρ_i_ of Ti and V in the supernatant from polished and textured Ti6Al4V in comparison with TCP indicates a release from the metal surfaces.

Multiple studies have investigated the consequences of metal released from implants regarding peri-implant diseases and implant failure [53]. According to Wachi et al. [54], titanium (Ti) ions released from the surface could influence the tissue degradation and stimulate the peri-implant mucositis in dental implants. A study from Wilson et al. [55] reports the occurrence of titanium (Ti) particles encapsulated by inflammatory tissue in 94% of the cases. Fretwurst et al. [56] identified metal particles in peri-implant soft tissue together with macrophages and the increase of titanium (Ti) within lymphocytes. Hence, the significantly lower titanium (Ti) mass concentration ρ_i_ on UV-LIPSS indicates a potential viable alternative for the reduction of peri-implantitis. It might be directly related to the lower cell LDH release C_L_ on UV-LIPSS.

Furthermore, it has to be noted that, in contrast to Ti release, Al release was higher from the modified surfaces if compared to PO. There is growing evidence of neurotoxicity of Al in humans and rodents [57,58]. Neurological disorders reported in dialysis patients are associated with high Al concentrations in the dialysate as well as with phosphate binding gels containing Al [59]. Additionally, different studies associated the presence of Al with the Alzheimer’s disease [60]. Al release and the resulting exposure and potential biological consequences have to be considered in the further course of preclinical testing.

#### 3.2.5. Osteogenic Differentiation

The presence of an osteogenic differentiation process enables the assessment of hMSCs ability to differentiate into osteoblasts and secreting mineral matrix (MM). Alkaline phosphatase (ALP) is an important component that supports the breaking down of key proteins for bone formation, which results on the cell mineral matrix (MM). The analysis comprehends the in vitro culture of hMSCs on TCP, PO, and UV-LIPSS. The samples textured within ultraviolet radiation were selected over the GR-LIPSS because of the promising results from previous experiments. The assessment includes the culture of hMSCs in expansion medium (EM) and osteogenic medium (OM).

**Figure 9:**
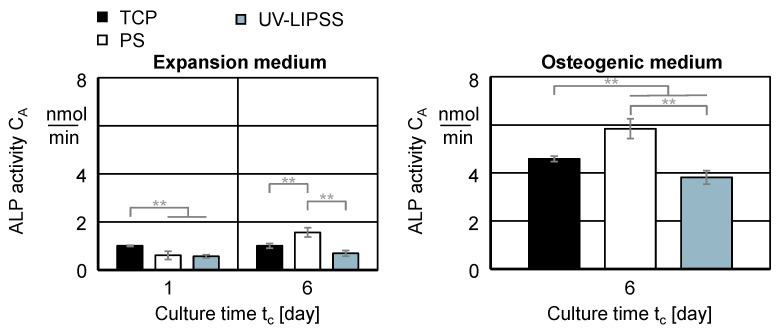
Cell ALP activity C_A_ on TCP, PO, and UV-LIPSS. Statistical analysis by ANOVA and Tukey’s test for the determination of statistically significant differences (n = 5 each group, ** for p < 0.01).

First, it assesses the cell ALP activity C_A_ on TCP, PO, and UV-LIPSS for the culture time t_c_ = 6 days within the culture medium EM and OM (Figure 9). The cell ALP activity C_A_ in EM is significantly higher for cells on TCP at t_c_ = 1 day and on PO at t_c_ = 6 days. The in vitro culture in OM at t_c_ = 6 days shows significantly higher ALP activity C_A_ on PO than TCP and UV-LIPSS. The outcomes of the textured surface is significantly lower than TCP. The ALP activity raises the levels of inorganic phosphate locally, which is a mineralization promoter. Furthermore, it also reduces the concentration of extracellular pyrophosphate, which is an inhibitor of mineral formation [61].

**Figure 10:**
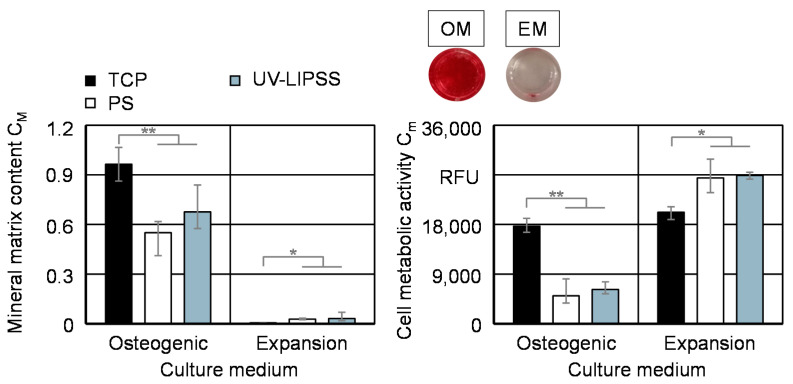
Cell mineral matrix content C_m_ on TCP, PO, and UV-LIPSS after culture time t_c_ = 16 days. Statistical analysis by ANOVA and Tukey’s test for the determination of statistically significant differences (n = 5 each group, * for p < 0.05, ** for p < 0.01).

Furthermore, the osteogenic differentiation assay comprises the cell mineral matrix content C_M_ and cell metabolic activity C_m_ for t_c_ = 16 days within OM and EM (Figure 10). The cell culture in OM foster the functionalization of the cells, allowing the differentiation and therewith the production of mineral matrix. The cell mineral matrix content C_M_ in the OM culture is significantly higher on TCP than on the metal surfaces. The cells cultured on UV-LIPSS present higher mean values of mineral matrix content C_M_, although not statistically significant. The low outcomes from the culture in EM indicates the lack of mineral matrix formation, which is expected since the culture media does not stimulate the osteogenic differentiation of hMSCs. The outcomes of cell metabolic activity C_m_ on TCP are significantly higher than PO and UV-LIPSS in OM. However, the opposite is observed by the culture in EM.

The ALP activity C_A_ comprehends an important osteoblast marker for differentiation. Although the in vitro culture on PO shows higher ALP activity C_A_, an increase of cell mineral matrix content C_M_ is inexistent. The outcomes show no significant difference between UV-LIPSS and the polished surface after the culture time t_c_ = 16 days. This outcome indicates that despite the lower ALP activity C_A_, the in vitro culture on UV-LIPSS offers an appropriate interface for osteogenic differentiation.

## 4. Conclusions

The current work investigates the laser surface texturing with low spatial frequency LIPSS (LSFLs) as a processing technique addressing the prevention of peri-implantitis. The structures manufactured presented spatial periodicity Λ inferior to the beam wavelength λ of the ultraviolet and green radiation. The preclinical in vitro assessment using hMSCs was essential to investigate the impacts of the proposed laser texturing on the surface biocompatibility as well as cytotoxicity.

Achieving laser surface texturing requires the arrangement of several laser tracks next to each other and overlapping of the tracks to ensure the continuity of the ripples over the entire surface. Thereby, the processing parameters of average fluence F_av_ and scanning speed v_f_ together with the lateral overlap rate Ψ play a major role for the LIPSS formation. Minor changes within the processing parameters result in discontinuities along the texturing. Melting spots or LIPSS discontinuities appear when the surface reaches the damage threshold. By insufficient energy E, non-textured spots remains on the surface compromising the profile symmetry.

The results showed that the hMSCs were capable of growing, proliferating, and differentiating on TCP, PO, and textured surfaces. The viability of cells was significantly higher for TCP in comparison with the metal surfaces. The cell metabolic activity C_m_ of PO, UV-LIPSS, and GR-LIPSS was similar during the entire culture time t_c_ = 9 days. PO presented a slightly higher cell proliferation C_p_ than UV-LIPSS and GR-LIPSS. However, the cytotoxic effects were significantly lower on UV-LIPSS considering the cell LDH release C_L_. Furthermore, the UV-LIPSS surface resulted in significantly lower release of Ti into supernatant, which was reported to have a correlation to the incurrence of peri-implantitis by several studies [55,56,62,63]. In contrast, Al release was higher in comparison to PO. One of the main mechanisms related with the toxic effects of metal nanoparticles is their ability to induce oxidative stress and mitochondrial dysfunction. Despite higher Al release from the textured surfaces, alterations of cellular metabolic activity were not observed in vitro. Therefore, a correlation between the Ti or Al release and mitochondrial dysfunction cannot be stated. Nevertheless, the potential involvement of systemic Al exposure in the pathogeneses of neurodegenerative diseases must be taken into consideration. The toxic potential of Al highly depends on its physico-chemical speciation. Thus further studies are necessary to characterize the in vitro and in vivo released Al. For osteogenic differentiation, cells cultured on the PO surface had significantly higher ALP activity CA. However, this did not result in higher cell mineral matrix content CM.

The outcomes of the in vitro experiments with primary hMSCs supports the hypothesis of this work. The surface texturing with UV-LIPSS shows promising in vitro biocompatibility. This is evidenced by the lower LDH release as well as lower titanium release if compared with the polished surface. However, these effects are not observed on GR-LIPSS. Future research activities should focus on exploring the potential of laser texturing of the manufacturing of sub-micron scale structures. The positive results from UV-LIPSS should be evaluated on other titanium alloys.

## Figures and Tables

**Table 1:** Nominal chemical composition of Ti6Al4V ELI alloy.

Chemical Elements	Al	V	Fe	O	C	N	Ti
Weight w (%)	6.11	3.93	0.12	0.11	0.01	0.01	Balance

**Table 2:** Laser processing parameters for manufacturing the laser-induced periodic surface structures (LIPSS).

Surface Texture	Average Fluence F_av_ (J/cm^2^)	Pulse Frequency f (kHz)	Scanning Speed v_f_ (mm/s)	Lateral Overlap Rate α (%)
UV-LIPSS	0.04	200.00	105.00	91.00
GR-LIPSS	0.10	200.00	150.00	82.00

**Table 3:** Surface characteristics of polished surface (PO) and textured surfaces.

Surface	Sa (nm)	Sz (nm)	Ssk (-)	Sku (-)	h_P_
PO	16 ≤ Sa ≤ 032	130 ≤ Sz ≤ 167	−0.04 ≤ Ssk ≤ −0.06	−0.09 ≤ Sku ≤ −1.29	-
UV-LIPSS	46 ≤ Sa ≤ 070	331 ≤ Sz ≤ 571	0.01 ≤ Ssk ≤ 0.20	0.38 ≤ Sku ≤ 0.58	56 ≤ h_P_ ≤ 89
GR-LIPSS	60 ≤ Sa ≤ 120	503 ≤ Sz ≤ 880	0.07 ≤ Ssk ≤ 0.29	0.03 ≤ Sku ≤ 0.63	56 ≤ h_P_ ≤ 92

**Table 4:** Quantitative results for the metallic elements in atomic percentage.

Element	PO	UV-LIPSS	GR-LIPSS
Ti(0)	4.53	4.29	
Ti(II)	4.29	3.99	2.81
Ti(IV)	69.39	73.68	58.78
Al(0)	1.44	2.34	
Al(III)	15.83	11.89	33.79
V(0)	0.46		
V(III)	1.18	2.30	1.16
V(IV)	1.83	1.52	1.93
V(V)	1.05	0.00	1.53

**Table 5:** Contact angle of water on PO and laser-textured surfaces.

Surface	Orientation to Ripples Direction	Contact Angle θ (°)	Spreading Coefficient |η| (-)
PO	-	49.1 ≤ θ ≤ 59.1	0.19
UV-LIPSS	Perpendicular	32.2 ≤ θ ≤ 37.5	0.22
UV-LIPSS	Parallel	35.1 ≤ θ ≤ 39.9	0.20
GR-LIPSS	Perpendicular	50.5 ≤ θ ≤ 58.5	0.18
GR-LIPSS	Parallel	55.4 ≤ θ ≤ 66.2	0.16
